# Effect of Rare Earth Metals (Y, La) and Refractory Metals (Mo, Ta, Re) to Improve the Mechanical Properties of W–Ni–Fe Alloy—A Review

**DOI:** 10.3390/ma14071660

**Published:** 2021-03-28

**Authors:** Senthilnathan Natarajan, Venkatachalam Gopalan, Raja Annamalai Arunjunai Rajan, Chun-Ping Jen

**Affiliations:** 1 School of Mechanical Engineering, Vellore Institute of Technology (VIT), Vellore 632014, India; senthil_nsn75@yahoo.co.in; 2 Centre for Innovation and Product Development, Vellore Institute of Technology (VIT), Chennai 600127, India; g.venkatachalam@vit.ac.in; 3 Centre for Innovative Manufacturing Research, Vellore Institute of Technology (VIT), Vellore 632014, India; raja.annamalai@vit.ac.in; 4 Department of Mechanical Engineering and Advanced Institute of Manufacturing for High-Tech Innovations, National Chung Cheng University, Chia-Yi 62102, Taiwan

**Keywords:** tungsten heavy alloy, refractory metal, rare earth element, microstructure, mechanical properties

## Abstract

Tungsten heavy alloys are two-phase metal matrix composites that include W–Ni–Fe and W–Ni–Cu. The significant feature of these alloys is their ability to acquire both strength and ductility. In order to improve the mechanical properties of the basic alloy and to limit or avoid the need for post-processing techniques, other elements are doped with the alloy and performance studies are carried out. This work focuses on the developments through the years in improving the performance of the classical tungsten heavy alloy of W–Ni–Fe through doping of other elements. The influence of the percentage addition of rare earth elements of yttrium, lanthanum, and their oxides and refractory metals such as rhenium, tantalum, and molybdenum on the mechanical properties of the heavy alloy is critically analyzed. Based on the microstructural and property evaluation, the effects of adding the elements at various proportions are discussed. The addition of molybdenum and rhenium to the heavy alloy gives good strength and ductility. The oxides of yttrium, when added in a small quantity, help to reduce the tungsten’s grain size and obtain good tensile and compressive strengths at high temperatures.

## 1. Introduction

Tungsten, as a metal, is unique due to its high melting point, high density, good thermal conductivity, and high elastic modulus [1]. In the early days of its application, tungsten was used in incandescent lamps in the mode of coils and wires. The application widened with the advent of new production and processing methods. The high melting point of tungsten makes it a premium element in plasma-facing components in reactors [2,3,4,5], electrical contacts [6], electron emitters, welding electrodes, sputtering targets, and heat sinks [7]. The other major feature of tungsten is its high density (19.3 g/cm^3^), which is a needed property in radiation shielding applications [8,9]. One major drawback of tungsten is its property of brittleness below the ductile-to-brittle transition temperature. This temperature varies depending on the preparation of tungsten. The poor ductility of tungsten poses a greater challenge in its performance in demanding applications. To improve the workability of tungsten, it is alloyed with other elements. The alloying additions are done to improve the dislocation mobility of atoms, to reduce the segregation of impurities along the grain boundaries, to refine the microstructure, and, thereby, to improve the strength and ductility of tungsten [10]. Over the years, alloying elements such as rhenium [11,12], niobium [11], vanadium, tantalum [13], molybdenum [14], copper [15], and oxides of lanthanum and yttria [16,17,18] have been investigated with tungsten.

The application of tungsten widened with the advent of tungsten heavy alloys (WHAs). The classical heavy alloy consists of tungsten-nickel-iron as one group and tungsten-nickel-copper as the other. The add-ons with tungsten help to maintain a distinctive combination of strength and ductility of the alloy. They are generally two-phase composites with a tungsten phase and a binder element phase [19]. The heavy alloys find application as counterweights, radiation shields, rotating inertia members like gyroscopes, semiconductor substrates, collimators, machining tools, kinetic energy penetrators, and fragmentation devices in defense [20]. Pure tungsten requires a very high sintering temperature, in the range of 1600 to 2000 °C, depending upon the tungsten particle size and the sintering method to get fully densified [21,22,23]. To overcome this difficulty in sintering and to introduce a ductile phase into the alloy, elements with a lower melting point and having preferably good solubility with tungsten are mixed with the base metal. The percentage of tungsten in the heavy alloy generally varies from 80 to 98 wt% [24,25]. The higher percentage of tungsten helps to maintain a higher density of the alloy. The powder metallurgy technique is primarily used to fabricate tungsten heavy alloys [26,27]. In a general process, the powders are mixed in a mixer or a ball mill to obtain a homogeneous mixture followed by compaction at a desired pressure in a press to obtain a green compact with sufficient strength. Further, the compact is sintered in a sintering furnace at a particular temperature and dwell time to get the final product with good density and strength [28]. During liquid phase sintering of W–Ni–Fe, the element with the lower melting point melts and the tungsten forms a solid solution with nickel and iron and, thereby, particle rearrangement takes place [29,30]. The addition of Ni improves the grain boundary activation of tungsten particles. The solubility of tungsten at a temperature of 1480 °C is about 45 and 30 wt% with nickel and iron, respectively [31]. In the liquid phase sintering of W–Ni–Fe, the alloy specimen may be subjected to shape distortion if the quantity of liquid phase is high. Hence, it is suggested to keep the liquid volume below 20% [32,33]. The iron and nickel are mutually soluble over a wide temperature range. The tungsten grains get dispersed in the face-centered cubic (FCC) matrix of Ni–Fe–W. The small grains dissolve in the matrix and, after saturation, get reprecipitated on coarser W grains. This process leads to microstructural changes and densification of the alloy. The addition of copper with tungsten does not provide good densification as both are mutually insoluble. However, with nickel as the main additive, the combination of W–Ni–Cu is able to produce a fully dense alloy [34,35].

Over the years, different sintering methods have been used to consolidate W–Ni–Fe and W–Ni–Cu heavy alloys. The sintering temperature for the liquid phase ranges from 1450 to 1500 °C. The conventional sintering method requires more sintering time to get a fully dense alloy. Using other sintering techniques, like microwave sintering and spark plasma sintering, the sintering time was reduced significantly. In the overall processing time, an 80% reduction was observed with the use of microwave sintering compared with the conventional method [36]. Spark plasma sintering is a unique technique where a pulsed DC current is supplied to the powder mixture simultaneously with the desired pressure to achieve a fully dense alloy in a significantly shorter sintering cycle [37]. It was also reported that in spark plasma sintering (SPS), oxide film formation was reduced and segregation of impurities was restricted due to surface activation of the powder particles [38]. Powder injection molding (PIM) is another processing method successfully used to produce tungsten heavy alloys [39,40]. Initially, the metal powders are mixed with an organic binder, usually a thermoplastic polymer, at a specific temperature and pressure and the resultant is known as the feedstock. The feedstock is then fed into the injection molding unit for processing to produce the mold, followed by debinding and sintering. It is understood that the PIM combines the metallurgy technique with the injection molding process. It is used for mass production of parts to produce dense and complex near-net-shape parts. The major challenge in the process is to maintain a defect-free shape of the product by properly controlling the debinding process. In recent years, new processing methods like laser melting deposition (LMD) [41,42] and selective laser melting (SLM) [43,44] have been attempted to produce tungsten heavy alloys. These additive manufacturing techniques are used to fabricate near-net-shaped parts of high complexity.

The mechanical properties of tungsten heavy alloys depend on various factors such as tungsten grain size, contiguity, matrix volume fraction, tungsten dissolution in the matrix phase, segregation of impurities on the tungsten matrix interface, intermetallic precipitation, and residual porosity [45,46,47]. The desired properties of strength and hardness can be obtained through swaging and heat treatment after sintering. However, the objective of obtaining near-net-shape components using the powder metallurgy technique without the need for further processing has not been met. The research over the years has been focused on improving the properties of the heavy alloy by avoiding thermo-mechanical treatments. One of the methods was to add other elements to the W–Ni–Fe to improve the strength of the alloy. Although studies are available on tungsten heavy alloys that discuss the processing methods and improvements in general, the specific analysis of the effects of alloying elements and their critical review is limited. This work investigates the alloying approaches to the elements rhenium, tantalum, and molybdenum and the oxide dispersion of yttrium and lanthanum on W–Ni–Fe alloys. The correlation between the microstructural evolution through different processing conditions and the mechanical properties of strength and ductility is examined. An understanding of the effects of alloying these elements in different proportions will help us to optimize the process of designing materials for specific requirements.

## 2. Effect of Rhenium and Tantalum on W–Ni–Fe

**Figure 1:**
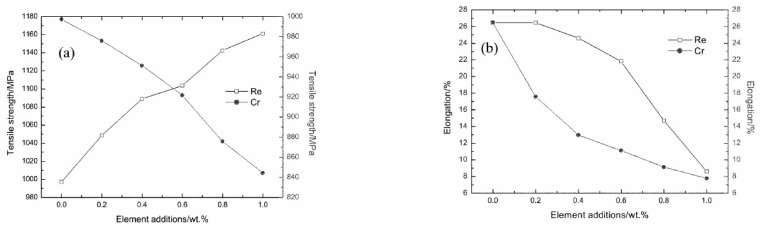
Variation in (**a**) tensile strength and (**b**) elongation with Re addition [50]. Reproduced with permission from Liu et al, Bulletin of Materials Science; published by Springer, 2008.

The investigations on the characterization of tungsten heavy alloy with additions of Rhenium (Re) and Tantalum (Ta) are limited. A positive outcome was observed in the case of the mechanical properties of the alloy with the addition of Rhenium in the range of 0.8–1.0 wt%. Re, in general, possesses good strength and toughness. It has a high melting point of 3182 °C but has good solubility in body-centered cubic (BCC) and FCC structures, leading to solid solution strengthening of the matrix and alloy. Earlier investigations by Bose and German [48,49] with Re addition revealed a good refinement of tungsten grains with an increase in the hardness and strength. However, there was a slight reduction in the percentage of elongation compared with the unalloyed system. The influence of 0.2 to 1 wt% of Re on 93W–4.9Ni–2.1Fe was investigated by Wensheng et al. [50]. The mixture was subjected to ball milling for 50 h, which resulted in a fine-grained alloy, followed by compaction and sintering at 1490 °C for a period of 90 min. The relative density and the tensile strength of the alloy showed an increasing trend with an increase in the amount of Re in the alloy as shown in Figure 1. The elongation was reduced as observed in the previous investigation [51]. However, the amount of reduction was only 15% with an increase in Re of up to 0.6 wt%. With a further increase in the additive, the rate of reduction in elongation increased.

**Figure 2:**
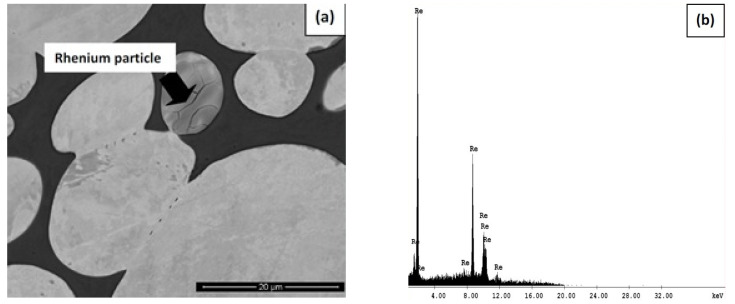
(**a**) SEM micrograph showing the presence of undissolved rhenium particles in the conventionally sintered alloy without milling and (**b**) Energy-Dispersive Spectroscopy pattern of rhenium (Re) particle [51]. Reproduced with permission from Ravi Kiran et al., Journal of Alloys and Compounds; published by Elsevier, 2017.

The strength of the alloy depends on the amount of rhenium dissolution in the matrix. If some of the rhenium particles are undissolved, they settle around the tungsten grains, leading to a decline in the properties of the alloy. High-energy milling of alloy powders was found to reduce the inhomogeneity in the alloy [49,51]. A novel method of producing the alloy was used by Ravi Kiran et al. [51]. The tungsten powder was mixed with 1 wt% of rhenium particles in a ball mill for 5 h. The milled powders were then mixed with nickel and iron particles and again milled for 48 h. The composition considered for the analysis was 89W–7Ni–3Fe–1Re. The sintering was done for 120 min at 1480 °C under a hydrogen atmosphere followed by heat treatment and oil quenching. The grain size got refined in the milled and sintered alloy to 18 microns compared with the 25 microns obtained with a conventionally sintered alloy without the milling process. However, the matrix volume fraction and tungsten dissolution in the matrix phase did not change significantly. However, the contiguity was reduced to 0.27 from 0.36 [51]. The microstructural parameters are shown in Table 1. The theory of undissolved rhenium particles is visible in the microstructure of the conventionally sintered alloy as shown in Figure 2. The milled alloy exhibited relatively good tensile strength. The mechanical properties obtained through various investigations are presented in Table 2.

**Figure 3:**
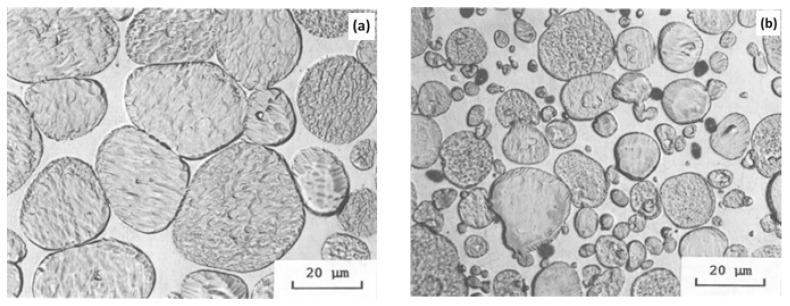
Microstructure of (**a**) 90W–7Ni–3Fe and (**b**) 85W–7Ni–3Fe with 5 wt% of tantalum, showing grain refinement and the presence of porosity [52]. Reproduced with permission from Bose and German, Metallurgical Transactions A; published by Springer, 1988.

Tantalum is a bcc-structured refractory material that has total solubility for tungsten and good dissolution in the matrix [52]. The investigations of tantalum-doped tungsten [53,54] revealed good refinement of tungsten grains. However, the presence of residual porosity and the brittleness of the structure at low and moderate temperatures limit the use of Ta with a tungsten alloy. A 5 wt% of Ta was added to the tungsten heavy alloy of 85W–7Ni–3Fe and sintered at a temperature of 1500 °C for 30 min in a hydrogen environment followed by heat treatment at 1100 °C for one hour. The microstructural difference between the alloy with tantalum and the alloy without tantalum was clearly visible as shown in Figure 3. The tantalum-doped alloy offers a refined grain size, but with the presence of some porosity. Although a high tensile strength (1025 MPa) is possible with this alloy, there is a significant drop in the elongation percentage, which is only 3% compared with the 31% obtained in undoped alloy.

## 3. Effect of Molybdenum on W–Ni–Fe

Molybdenum is added to the tungsten heavy alloy of W–Ni–Fe to reduce the dissolution of tungsten in the matrix phase and thereby refine the grains, leading to good mechanical properties. The grain growth reduction is required in order to get a high-strength alloy.

### 3.1. Sintering Mechanism of W–Ni–Fe-Mo Alloy

As the sintering progresses, the liquid phase forms early in the heavy tungsten alloy with added molybdenum in comparison with the base W–Ni–Fe heavy alloy. This is due to the fact that molybdenum combines with nickel to form a eutectic liquid at around 1320 °C. However, tungsten takes a longer time to form the liquid phase with nickel, which happens at 1455 °C [55,56]. Hence, the dissolution of tungsten in the matrix phase is restricted in the early phase of sintering. However, molybdenum starts to dissolve in the matrix due to the early formation of the liquid phase. So, at the early stage of sintering, the microstructure is a combination of tungsten grains and a matrix of Ni–Fe–Mo with a little dissolution of tungsten in the matrix [57]. Therefore, the grain growth in the initial stage of sintering of the WHA with added molybdenum is less than the corresponding growth in pure WHA.

During the further progression of the liquid-phase sintering process, the molybdenum gets partitioned between the matrix of Ni–Fe–W and the tungsten-rich grains. It has been reported that the total solubility of W + Mo is constant at a given temperature [57]. The molybdenum and tungsten possess complete solubility in all compositions and at any temperature. The grain growth mechanism at the sintering hold is referred to as the solution and re-precipitation process, where large tungsten grains grow and small grains get dissolved and re-precipitated over the grain boundaries. The precipitation composition at this stage is the combination of W–Mo in the alloy. Initially, the concentration of molybdenum is higher in the precipitation. This leads to a decrease in the molybdenum content in the liquid phase. Now, the tungsten dissolution in the liquid phase increases since the total concentration of tungsten and molybdenum is relatively constant in the liquid [58]. At a certain point, the liquid composition reaches a constant stage and further sintering does not contribute much to the densification of the alloy.

### 3.2. Microstructural Analysis of W–Ni–Fe-Mo

**Figure 4:**
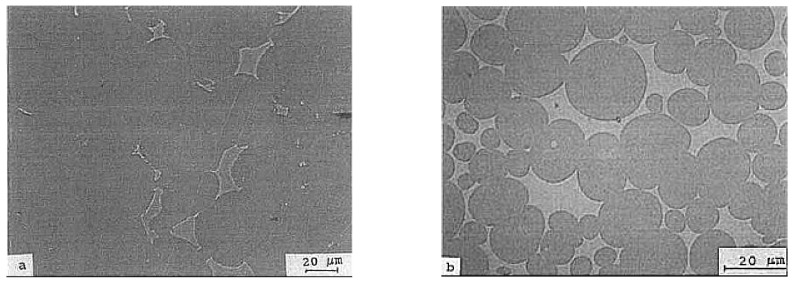
Microstructure of 82W–8Mo–8Ni–2Fe sintered at 1500 °C for 480 min: (**a**) slowly cooled; (**b**) water-quenched [57]. Reproduced with permission from Kemp and German, Journal of the Less Common Metals; published by Elsevier, 1991.

In the WHA with added molybdenum, the controlling factors are the isothermal hold during sintering and the cooling rate. A slow cooling rate and a high sintering temperature hold lead to the formation of different phases and intermetallic compounds [57,59]. Kemp and German [57] studied these effects on 82W–8Mo–8Ni–2Fe alloy prepared using the injection molding method and sintered at 1500 °C for holding times of 30, 120, 480, 960, and 1920 min followed by slow cooling. Smooth, rounded grain structures were obtained for the alloy sintered for 960 min. However, the alloy sintered for 480 min showed a similar microstructure when it was water-quenched instead of slow-cooled as shown in Figure 4. The slowly cooled alloy exhibited many phase reactions, which were found to exist even after the standard post heat treatment process (1100 °C for 60 min). Hence, proper control of the cooling rate is required to obtain an alloy with good mechanical properties.

**Figure 5:**
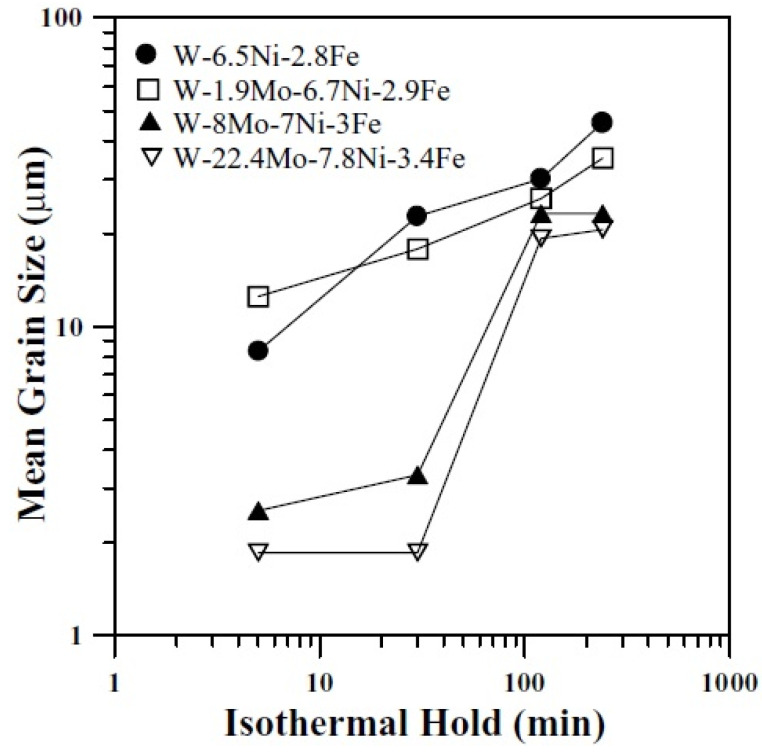
Variation in mean grain size with sintering holding time [60]. Reproduced with permission from Hsu et al., Journal of Materials Science; published by Springer, 2003.

The effect of different proportions of molybdenum addition on the tungsten grain growth was investigated by Hsu and Lin [60]. The sintering profile followed for these alloys of W–6.5Ni–2.8Fe, W–1.9Mo–6.7Ni–2.9Fe, W–8Mo–7Ni–3Fe, and W–22.4Mo–7.8Ni–3.4Fe had an intermittent isothermal hold at different temperatures. At 350 °C and 500 °C, there was an isothermal hold of 60 min and at 1000 °C again the hold was for 60 min. Finally, at 1500 °C, the sample was held for 5, 15, 30, 60, 120, 180, or 240 min. The thermal profile was attributed to dewaxing, reducing oxides, and, finally, densifying the alloy. The first liquid phase for the alloys occurred at a different range of temperatures. As observed previously, in alloys with added molybdenum the first liquid formation occurs earlier compared with the classical heavy alloy. The different kinds of microstructures obtained for the sintered alloys infer different kinds of phase partitioning mechanisms occurring in the alloys. The structure not only depends on the amount of molybdenum in the alloy but also on the duration of the sintering hold. Figure 5 shows the effect of sintering holding time on the grain size. The alloys with 8 Mo and 22.4 Mo showed a smaller grain size up to an isothermal hold of 30 min. After this, the grain growth increases at a faster rate, attributed to the instability in the structure causing smaller grains to disappear. It was observed that excess Mo concentration does not contribute to the grain refinement at extended isothermal holds. Further, it also leads to the precipitation of intermetallic phases during cooling [61]. A novel way of producing the W–Ni–Fe-Mo alloy was attempted by Lin et al. [62] using separate thin Mo layers over the W–Ni–Fe alloy surface, which diffuses into the alloy during sintering. The Mo atoms diffused into the tungsten grains and binder phase, but the concentration of Mo varied along different sections of the alloy.

### 3.3. Mechanical Properties of W–Ni–Fe-Mo

The addition of molybdenum to the tungsten heavy alloy increases the strength of the alloy. Table 3 shows the hardness, tensile strength, and percentage elongation of the alloys under specific processing conditions. With an increase in the molybdenum content, the hardness of the alloys was found to have an inverse correlation with ductility. Bose and German [63] considered the composition of the alloys with 7/3 and 8/2 ratios of Ni/Fe. It was observed that the hardness and tensile strength significantly increased when compared with the conventional heavy alloy of W–Ni–Fe without molybdenum. However, the increase in molybdenum concentration decreased the ductility of the matrix phase. The scrutiny of the results shows that the decrease in ductility can be restricted to some extent with the use of the 8/2 ratio of Ni/Fe, probably due to the fact that it slightly improves the tungsten solubility in the matrix [52].

Sintering of the alloys at lower temperatures was attempted using the spark plasma sintering process [64,65]. The heating rate of the spark plasma sintering process is very high, usually at a rate of 100 °C/min. The alloy W–2Mo–7Ni–3Fe was sintered in SPS in a temperature range of 1000–1250 °C [66] and the microstructure and mechanical properties were investigated. The process provided a refined W-grain size with a maximum of 5 microns. The highest bending strength obtained was 390 MPa. However, the fracture modes showed more pores and intergranular fracture of W-grains, which implies lower ductility of the binder phase. Another investigation using SPS of the alloy with a 7:3 Ni/Fe ratio and varying the Mo content from 0 to 16 wt% and sintering at 1100 °C also yielded good tensile strength and hardness for the alloys [65]. The spark plasma sintering of the alloy with the Ni/Fe ratio maintained at 8:2 was analyzed by Prasad et al. [66]. The molybdenum addition was varied from 0 to 24 wt% and sintered at 1000 °C. The mixed powders were subjected to high-energy ball milling for 40 h under an argon atmosphere before sintering. The density was found to increase with the increase in molybdenum content and was highest for 24 wt% of molybdenum. A similar trend was observed for the tensile strength and hardness. However, the percentage elongation reduced from 28 to 2%.

## 4. Effect of Oxide Dispersions on WHA

The tensile and impact properties of the tungsten heavy alloy also depend on the segregation of impurities like H, P, S, C, and O over the W and matrix interface [67,68]. This causes embrittlement in the alloy, leading to reduced mechanical properties. Based on the percentage of segregation of impurities, the bonding strength between the tungsten and the matrix is altered and the continuity of the matrix is affected, leading to fracture. Some elements like H can be removed by heat treatment. The dispersion technique is used to lower the effect of embrittlement by the formation of stable compounds, which depend on the type of dispersion used.

### 4.1. Effect of Lanthanum and Lanthanum Oxide on W–Ni–Fe

The creep strength and recrystallization temperature of tungsten materials can be improved by dispersing oxides of lanthanum or yttria [54]. The addition of lanthanum to steel successfully reduced the temper embrittlement that occurs due to P and S [69,70]. This is the impetus for experimenting with oxides of lanthanum with tungsten heavy alloys.

#### Microstructure Analysis and Mechanical Properties

**Figure 6:**
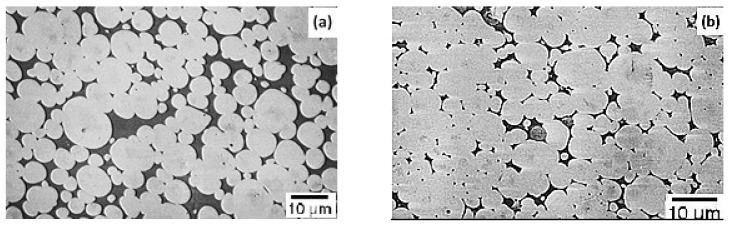
Microstructure of the W–Ni–Fe-Mo alloy (**a**) without La and (**b**) with 0.4% La [71]. Reproduced with permission from Wu et al., International Journal of Refractory Metals and Hard Materials; published by Elsevier, 1999.

**Figure 7:**
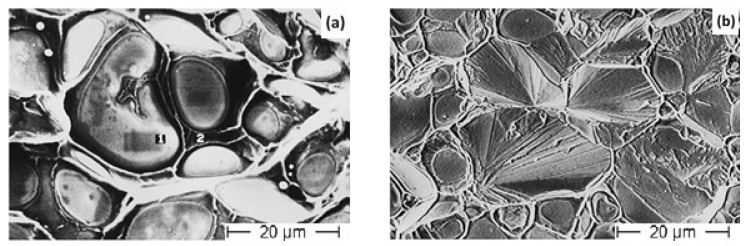
Fracture surfaces of the W–Ni–Fe-Mo alloy (**a**) without La and (**b**) with 0.4% La [71]. Reproduced with permission from Wu et al., International Journal of Refractory Metals and Hard Materials; published by Elsevier, 1999.

Wu et al. [71] investigated the microstructure and mechanical properties of 78W–2.5Ni–2.0Fe alloy with the addition of approximately 17 wt% of molybdenum, varying proportions of lanthanum (0.2–0.8 wt%), and 0.2 wt% of Mn. The powder mixture was cold iso-statically pressed and sintered under a hydrogen atmosphere at 1510 °C for 90 min. The microstructure of the alloys with added La is typical to that of a classical tungsten heavy alloy as shown in Figure 6. In addition to the W and the matrix phase, there exists a secondary phase with La as the major component. From the X-Ray Diffraction (XRD) pattern, the phases were identified as LaMnO_3_ and Mn_3_O_4_. The phases were stable in nature. This reduces the segregation of the oxygen element on the W-matrix interface and enhances its bonding. The tensile strength increases from 291 MPa for the alloy without La addition to 903 MPa for the alloy with 0.4 wt% La. The elongation also increases to 4.7%. The fracture surface analysis also supports the data. The fractography of the 0.4% La alloy revealed a ductile fracture with a rupture in the matrix phase as shown in Figure 7. However, for the alloys with a higher percentage of La (0.6 and 0.8 wt%), the tensile strength and elongation were observed to decrease. This change in trend was attributed to the formation of more La-rich phases, which leads to the discontinuity in the matrix.

**Figure 8:**
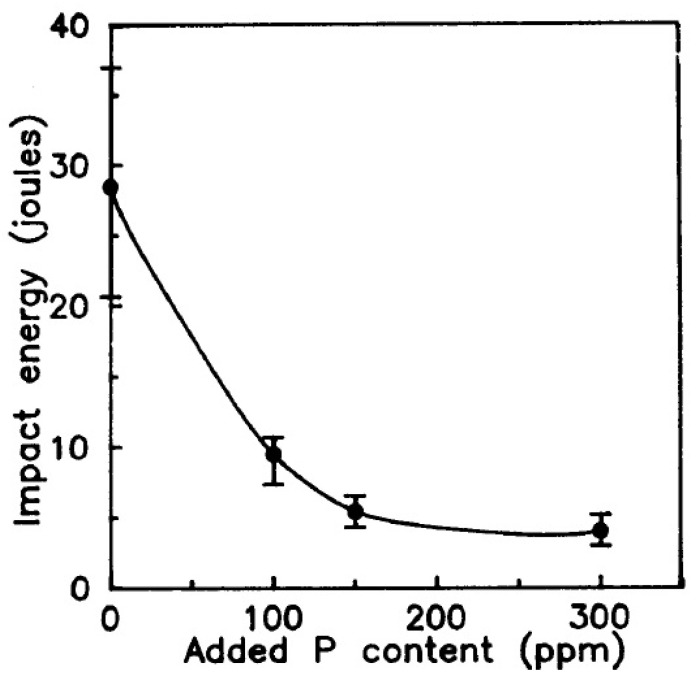
Effect of P on the impact energy of 93W-4.9Ni-2.1Fe [67]. Reproduced with permission from Hong et al., Metallurgical Transactions A; published by Springer, 1991.

**Figure 9:**
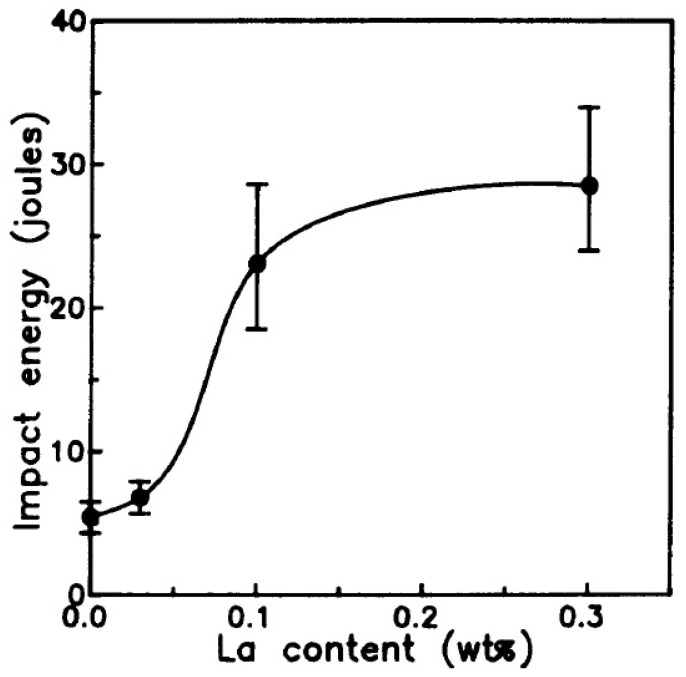
Effect of La on the impact energy of the alloy doped with 150 ppm of P [67]. Reproduced with permission from Hong et al., Metallurgical Transactions A; published by Springer, 1991.

The effect of La on reducing the embrittlement caused by the phosphorous element was investigated by Hong et al. [67]. A higher concentration of phosphorous leads to a sharp decrease in the impact strength of the alloy as evident from Figure 8. This is evident with the amount of phosphorous present in the fractured surfaces of the alloy subjected to an impact test. In the work by Hong et al., lanthanum was added to the 93W–4.9Ni–2.1Fe alloy to study its influence on limiting the embrittlement effect of phosphorous. The W powder contained 19 ppm of phosphorous. For a better analysis, the alloy was also doped with 150 ppm of P and lanthanum was added at the percentage of 0.03, 0.1, and 0.3. The La addition did not reveal any significant improvement in the ultimate tensile strength and hardness of the alloy in comparison with the undoped specimen. There was a slight reduction in the elongation from 20 to 17%. However, the impact energy of the alloy with La addition showed a substantial increase with the increase in the percentage of La. The variation in impact energy with respect to La addition is shown in Figure 9. The La in association with O and P is expected to form a stable compound in the form of LaPO_4_. Additionally, it is stated to form another stable compound of La_2_O_3_ [72,73]. Thus, the La addition was found to reduce the segregation of phosphorous at the interfaces for the alloys with a relatively higher concentration of P and under slow cooling conditions after heat treatment.

**Figure 10:**
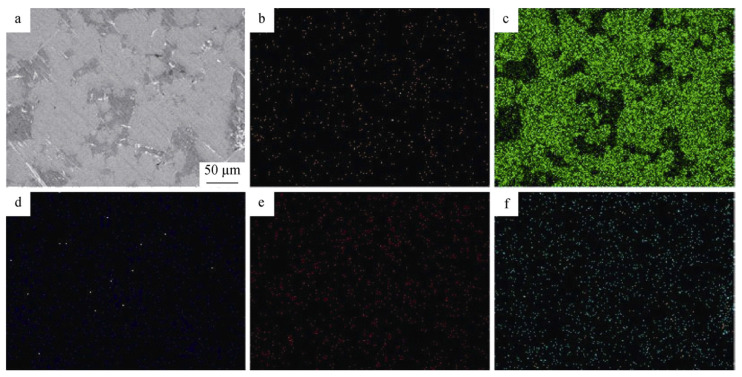
Elemental distribution of SEM micrograph on (**a**) spark-plasma-sintered W-7Ni-3Fe-0.5La_2_O_3_; (**b**) Ni; (**c**) W; (**d**) Fe; (**e**) La; and (**f**) O [75]. Reproduced with permission from Muthuchamy et al., Rare Metals; published by Springer, 2020

The addition of lanthanum oxide to the tungsten material was found to improve the fracture strength at room temperature after high-temperature annealing. The recrystallization temperature was also improved [74]. The tungsten alloy with La_2_O_3_ added gave a better performance than the undoped specimen. The behavior of the lanthanum-oxide-dispersed tungsten heavy alloy under high heating rate conditions was investigated by Ayyapparaj et al. [75]. To avoid extended exposure of the alloy to a high temperature during sintering and to reduce the implications, spark plasma sintering was used, which is a high-rate sintering process. The tungsten alloy considered in the experiment was W-7Ni-3Fe. The amount of La_2_O_3_ addition was varied from 0.25 to 1.00 wt%. The sintering parameters included a temperature of 1100 °C, a heating rate of 100 °C/min, and a pressure of 30 MPa. The density of the oxide-dispersed heavy alloy was more than that of the undoped heavy alloy. The La_2_O_3_ particles got distributed uniformly over the W grains and the matrix phase, which contributed to the matrix strengthening effect. The elemental distribution is shown in Figure 10. Thus, the tensile strength and hardness of the alloy increased with the increase in the concentration of the lanthanum oxide, resulting in an alloy with good strength relative to the undoped one. A high tensile strength of 1110 MPa was obtained for the alloy with 1 wt% La_2_O_3_ added. The tungsten grain size also got refined to 7.89 microns. This was due to the effect of the oxide dispersion and the high-temperature sintering process. However, the percentage elongation of the alloy decreased with the increase in the oxide dispersion of the lanthanum due to the brittleness induced in the alloy [76,77]. The mechanical properties of the heavy alloy with lanthanum addition are presented in Table 4.

### 4.2. Effect of Yttrium and Yttrium Oxide on W–Ni–Fe

**Figure 11:**
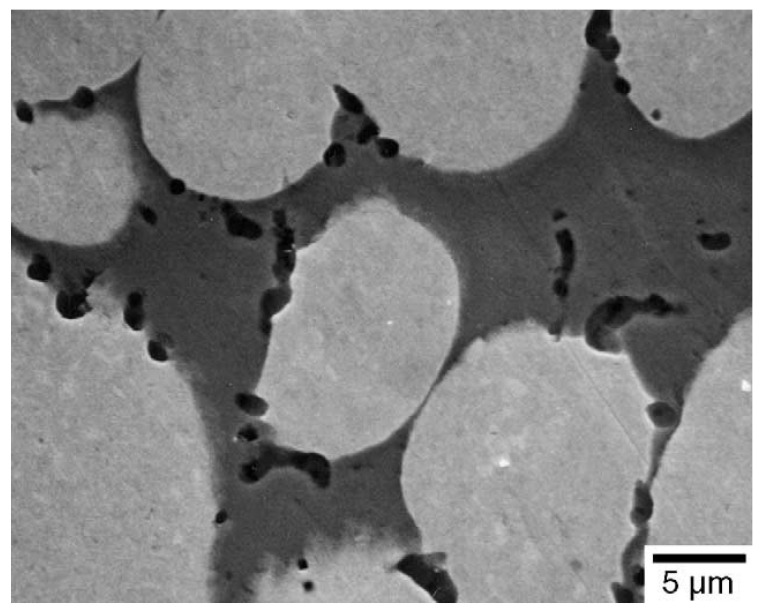
SEM micrograph of a mechanically alloyed oxide dispersion of 1 wt% Y_2_O_3_ in W-5.6Ni-1.4Fe sintered at 1485 °C for 60 min [80]. Reproduced with permission from Ryu et al., Materials Science and Engineering: A; published by Elsevier, 2003.

**Figure 12:**
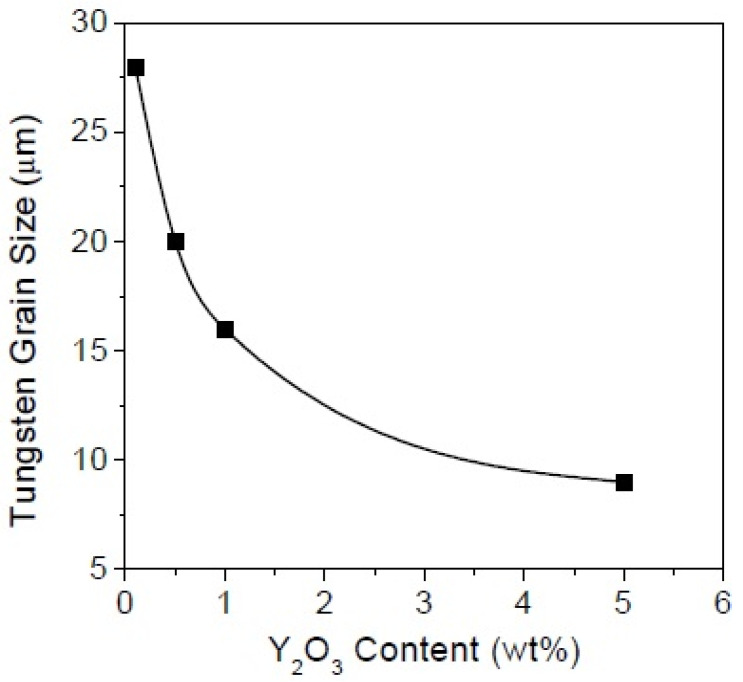
Variation in grain size in mechanically alloyed and sintered W-5.6Ni-1.4Fe with increasing Y_2_O_3_ content [80]. Reproduced with permission from Ryu et al., Materials Science and Engineering: A; published by Elsevier, 2003.

Yttrium is another rare earth element used in the production of tungsten heavy alloys to improve its properties. Early investigations of yttrium-oxide-dispersed tungsten material showed good corrosion resistance over the grain boundaries against molten metals [78,79]. The grains got refined with the addition of Y_2_O_3_ in comparison with pure tungsten. The effect of sintering temperature on the addition of 5, 10, and 20 vol% of yttrium oxide to tungsten was studied. The relative density increased with the increase in the volume of the oxide particles. The Y_2_O_3_ particles grew larger in size with the increase in the temperature from 1800 to 2200 °C. However, the bending strength decreased with increasing sintering temperature. The Y_2_O_3_ oxide dispersion on the tungsten heavy alloy showed a similar behavior of refining the tungsten grain size with an increase in the content of the oxide at a given sintering temperature and time [80]. The heavy alloy of 93W–5.6Ni–1.4Fe was alloyed with 0.1, 0.5, 1, and 5 wt% of Y_2_O_3_ powder particles in a ball mill for 72 h. After compaction at 100 MPa, the green compacts were sintered at a temperature of 1485 °C for 60 min in a hydrogen atmosphere. The oxide particles got widely distributed on the tungsten–matrix interfaces, thereby restricting the dissolution of tungsten in the matrix phase and resulting in refinement of the tungsten grains [81]. Figure 11 shows the distribution of the Y_2_O_3_ particles in the alloy. The tungsten grain size reduced drastically from 27 microns to 10 microns with the increase in the amount of oxide in the heavy alloy. This positive trend is represented in Figure 12. To understand the mechanical behavior of the alloys, they were subjected to a tensile test and a high-temperature compression test. There was a slight decrease in the tensile strength and elongation for the alloy with 0.1 wt% Y_2_O_3_ added (828 MPa and 14.6%, respectively) compared with the undoped alloy (940 MPa and 30%, respectively). This was attributed to the difference in the relative densities of the alloys. However, relatively better strength and elongation were obtained for the doped alloy when it was sintered for 120 min with an increase in density.

The compression test was carried out at 800 °C to study the deformation behavior of the alloy at elevated temperatures. The test showed a satisfactory result with an improvement in the compression strength as the oxide content was increased. The alloy was able to maintain the oxide dispersion strengthening effect even at high temperatures. Another investigation by Fan et al. [82] with an yttrium-oxide-dispersed tungsten heavy alloy showed good tensile strength as well as good ductility with the addition of a low percentage of the oxide. Fine-grained alloys were prepared using a mechanical alloying process by dispersing 0.02–0.08 wt% of Y_2_O_3_ into 90W–7Ni–3Fe in a ball mill for 40 h at 200 rpm, which resulted in composite powder with a size of 21.4 nm. The sintering was done at 1480 °C for 30 min under a hydrogen atmosphere. All the oxide-dispersed samples showed a relative density of more than 99%. Generally, nano powder particles are prone to bubble formation during liquid phase sintering, which will lead to a lower density for the alloys. However, small amount of oxide dispersion inhibits the bubble production and enhances the density. The alloy with 0.04 wt% Y_2_O_3_ remarkably gave a high tensile strength of 1050 MPa as well as a good ductility of 30%. Generally, tensile strength and ductility have an inverse correlation. However, the effective performance of this alloy may be due to its fine-grained structure and the homogeneous distribution of the oxide phase. The oxides absorb the impurities and oxygen and prevent them from segregating over the tungsten–matrix interface. A high temperature tensile test in the range of 25–1100 °C on a fine grained 93W–4.9Ni–2.1Fe–0.03Y alloy showed a superior ultimate tensile strength to the test on the coarse-grained alloy of 93W–4.9Ni–2.1Fe [83]. A model alloy of W–Ni–Fe-Mo-Co was chosen by Chen et al. [84] to investigate the mechanical properties of the sintered alloy with and without yttrium oxide dispersion. The dispersed alloy showed a relatively high hardness of 430 HV, giving a 17% increase in hardness compared with the undispersed alloy. The mechanical properties of some oxide-dispersed tungsten heavy alloys are presented in Table 5.

**Figure 13:**
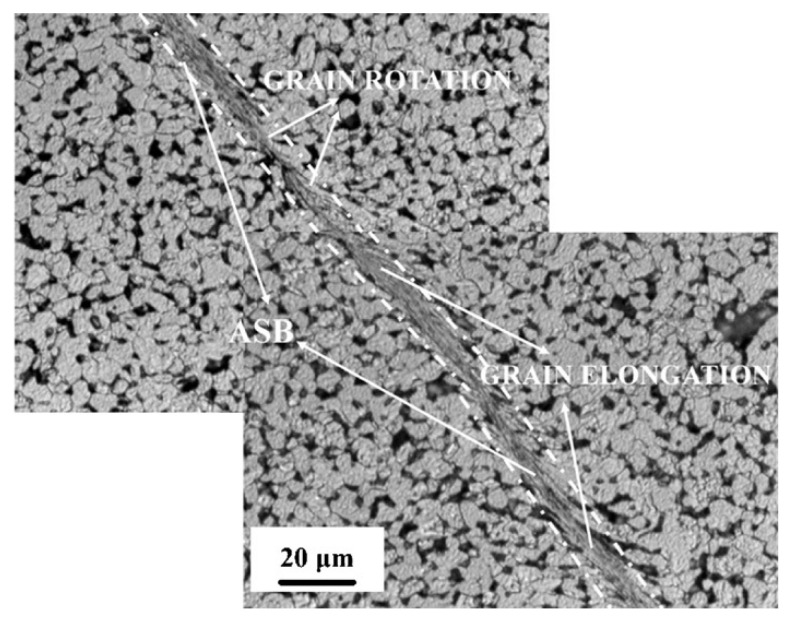
The adiabatic shear band is visible in the specimen dynamically tested at a strain rate of 1.9 × 10^3^ s^−1^ [87]. Reproduced with permission from Gong et al., Materials Science and Engineering: A; published by Elsevier, 2010.

The tungsten heavy alloys were found to be a replacement for Depleted Uranium (DU) alloys in military applications, especially as armor-piercing penetrators. However, the performance of WHAs is not on par with DU alloys at high strain rates [85,86]. High density and good penetration characteristics are required to obtain an alloy with good performance for armor application. The characteristics include formation of a localized shear band at low strains and a reduction in the thermal softening effect. Several works on enhancing the adiabatic shear failure of coarse-grained tungsten heavy alloys have been carried out over the last decade. Gong et al. [87] studied the dynamic behavior of a fine-grained alloy of 93W–4.9Ni–2.1Fe–0.03Y in the strain range of 1200 to 1900 s^−1^. The shear band occurred in the alloy tested at the strain rate of 1900 s^−1^ and is represented in Figure 13. The grains elongate in the band region due to the intensive localized plastic flow. The adiabatic shear failure in the fine-grained alloy seems to occur not due to the thermal softening as in the case of coarse-grained alloys but due to the premature shear localization. The effect may be attributed to fine grains and the increase in the number of grain boundaries, which reduces the rate sensitivity and strain hardening [88].

**Figure 14:**
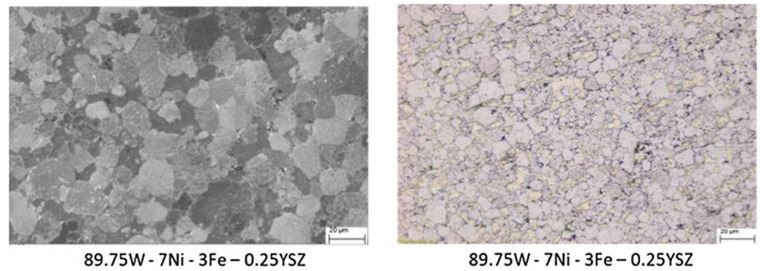
The SEM and optical micrographs of SPS-sintered alloy with 0.25 wt% yttria-stabilized zirconia (YSZ) [89]. “Reproduced with permission from Muthuchamy et al., Arabian Journal for Science and Engineering; published by Springer”.

A novel tungsten heavy alloy was developed by Muthuchamy et al. [89] by dispersing yttria-stabilized zirconia (YSZ) into a W–Ni–Fe alloy with a 7:3 nickel–iron ratio. The alloy was sintered in SPS at 1100 °C. The amount of dispersed particles varied from 0 to 1.0 wt%. The sintered density decreased with an increase in YSZ content. The increase in the oxide phase resulted in the reduction of the binder phase, leading to a decrease in density. However, the density obtained for the doped alloys was higher than that obtained for the undoped one. The optical and SEM micrographs for the 0.25 wt% doped alloy as shown in Figure 14 reveal good dispersion of the oxide phase. The tensile strength and hardness were also higher for this alloy. However, the ductility for all the alloys was very low, which may have been due to the restrictions in the dislocation movements along the tungsten–matrix interface due to the presence of more aggregates.

## 5. Discussion and Conclusions

The rare earth metals, like yttrium, lanthanum, and their oxides, and refractory metals, like molybdenum, tantalum, rhenium, and niobium, are effective in refining the tungsten grain size of the tungsten heavy alloy of W–Ni–Fe by restricting the tungsten dissolution in the liquid phase during sintering of the alloy. Grain size is a significant factor in obtaining a good yield strength of the alloy. With a reduced grain size, the probability of moving the dislocations formed at the boundaries to induce plasticity is lower [90]. This is known as the Hall-Petch strengthening effect, which results in a superior yield strength for the alloy. The yield strength and grain size have an inverse correlation as expressed by Hall and Petch [91]. To get better mechanical properties and homogeneity, the investigators are more interested in the processing of nano-sized compositions of the tungsten heavy alloy. This is obtained through high-energy ball milling of the as-purchased powder particles for several hours. Particle sizes in the range of 4 to 26 nm can be produced through this process. The major issue with this technique is the contamination of the powders with the milling medium of the balls and the atmosphere. This leads to segregation of impurities on the tungsten boundaries and formation of intermetallic precipitates during the sintering process. Some of the techniques, like using alloy-coated milling media with a protective atmosphere and using surfactants, can reduce the contamination effect [92,93].

The major requirement is to obtain an alloy with good strength without a significant drop in ductility. The addition of the rhenium element positively satisfies this requirement as can be seen from the investigated data presented in Table 2. However, the high cost of the rhenium element limits its usage as an effective additive for tungsten heavy alloys. Now, since the element cost is reduced due to the increase in rhenium recycling and the drop in demand for the element in catalysts, more investigations using rhenium can be expected in the near future. The factor to be controlled in the processing of rhenium–tungsten heavy alloys is the presence of undissolved rhenium particles over the tungsten phase. The Re-rich regions may become crack initiation spots, leading to a reduction in the alloy’s strength. Tantalum is not used commercially with tungsten alloys because of the brittleness of the structure in a wide temperature range and the formation of residual pores during the sintering process. Although it provides a high tensile strength [54] for the alloy, the ductility of the matrix in terms of percentage elongation is reduced to a very low value.

Molybdenum is another promising refractory element that provides a tungsten heavy alloy with good strength and sufficient ductility, as it is softer and more ductile than tungsten. Up to 8 wt% addition results in a unique combination of good tensile strength and ductility. A further increase in the content leads to a further drop in the ductility of the alloy. The drop in percentage elongation can be controlled with the use of a 8/2 Ni/Fe ratio in the alloy [63]. The Mo doping decreases the melting point of the tungsten heavy alloy and yields fine grains. The important factors that have to be controlled while processing W–Ni–Fe-Mo alloys are the duration of the isothermal sintering hold and the cooling rate. A higher sintering hold duration and a slow cooling rate lead to the formation of intermetallic phases and a drop in the mechanical properties of the alloy.

The oxide dispersion of yttrium or lanthanum is used to reduce the segregation of impurities on the tungsten–matrix interface by forming stable compounds. Better results are possible with a lower percentage of oxide dispersion. When the rare earth oxide content is very high, the remaining oxides after the formation of compounds re-precipitate in the tungsten–matrix interface. This prevents the migration and diffusion of atoms during liquid-phase sintering of the alloy, leading to a lower density. To overcome this drawback, the sintering time has to be increased. However, this may lead to an increase in the tungsten grain size. Hence, optimum use of the oxide content is necessary to get the desired properties of the alloy. At high temperatures, the tensile strength and compression strength get increased with the oxide dispersion technique. In the application of armor plate piercing penetrators, the material should have self-sharpening susceptibility during deformation at high strain rates. The mechanically alloyed yttrium-oxide-dispersed tungsten heavy alloy was found to satisfy the penetration requirement as experimented with and patented by Hong et al. [94]. The amount of tungsten should be maintained at 90 wt% and above to get a high-density penetrator of more than 17 g/cm^3^ [83]. The oxide-dispersed alloy showed a larger piercing depth per density compared with the undoped tungsten heavy alloy of W–Ni–Fe. The addition of yttrium is more effective in maintaining the ductility of the alloy compared with the lanthanum oxide dispersion. The brittle nature of lanthanum reduces the percentage elongation with an increase in the content of the oxide dispersion. However, it is more effective at controlling the phosphorous content in the alloy. When the content of P is higher in the alloy, the impact strength is reduced. The La addition effectively reduces this detrimental effect and enables the impact strength to increase with an increase in La content. Experiments were also performed with the addition of Al_2_O_3_, but the results were not promising. It did not show any improvement in the mechanical properties of the alloy. Instead, it resulted in a 15% reduction in the tensile strength and a huge reduction (from 21% to 3%) in elongation compared with the undoped alloy of 93W–4.9Ni–2.1Fe [95]. More pores were visible in the doped alloy, which showed a good compression strength at an elevated temperature as is usually seen for oxide-dispersed alloys [80].

The discussions lead to the following conclusions:

- The strength and hardness of tungsten heavy alloys depend on the bonding strength of the W–matrix interface. A W–Ni–Fe alloy with good strength is made possible with the addition of rhenium and molybdenum elements.
- The correlation between the microstructural parameters and mechanical properties shows that a smaller grain size is an effective factor in improving the strength and ductility of the tungsten heavy alloy. The drop in ductility in a molybdenum-added heavy alloy can be controlled to some extent with the use of a 8/2 ratio of Ni/Fe.
- The oxide dispersion of yttrium with W–Ni–Fe is found to be more effective in obtaining an alloy with good strength and ductility compared with a lanthanum oxide dispersion. A fine-grained alloy with yttrium dispersion leads to a microstructure that can promote adiabatic shear banding, which is a necessary criterion for kinetic energy penetrator applications.
- Processing techniques like spark plasma sintering are observed to produce tungsten heavy alloys with higher yield and tensile strength. The optimum sintering temperature and time are also important factors to be controlled in obtaining an alloy with specific mechanical properties.
- Developments in processing methods like additive manufacturing techniques will help to improve the alloying techniques with tungsten heavy alloys and to produce complex net-shaped parts with higher strength.

## Figures and Tables

**Table 1:** Microstructural parameters of the Re added to the tungsten heavy alloy [51].

Alloy Type	Tungsten Grain Size (µm)	Matrix Volume Fraction (%)	Contiguity	Dihedral Angle (°)
Conventional	25 ± 11	17 ± 3	0.36 ± 0.07	58 ± 1
Milled	18 ± 6	19 ± 1	0.27 ± 0.06	42 ± 2

**Table 2:** Mechanical properties of the rhenium and tantalum added to the tungsten heavy alloy of W–Ni–Fe.

Alloy	Sintering Process	Hardness (HRA)	Tensile Strength (MPa)	Elongation (%)	Reference
W–8Ni–2Fe–6Re	Sintering at 1500 °C for 60 min	—	1180	13	[49]
W–4.9Ni–2.1Fe	Ball milled for 50 h Sintered at 1490 °C for 90 min	—	997	26.4	[50]
W–4.9Ni–2.1Fe–0.2Re		—	1050	26	
W–4.9Ni–2.1Fe–0.4Re		—	1090	25	
W–7Ni–3Fe–1Re	Sintering at 1480 °C for 120 min followed by heat treatment	—	890	14	[51]
W–7Ni–3Fe–1Re	W–Re milled for 5 h followed by 48 h of milling of the whole mixture. Sintering at 1480 °C for 120 min followed by heat treatment	—	952	23	
W–7Ni–3Fe	Sintering at 1500 °C for 30 min and heat treated at 1100 °C for one hour	62.8	925	31	[52]
W–7Ni–3Fe–5Ta		69	1025	3	

**Table 3:** Mechanical properties of tungsten heavy alloy of W–Ni–Fe with added molybdenum.

Alloy	Sintering Process	Hardness (HRA)	Tensile Strength (MPa)	Elongation (%)	Reference
W–7Ni–3Fe	Conventional sintering 1500 °C for 30 min Heat treated at 1100 °C for 1 h and water-quenched	62.8	923	30	[63]
W–8Ni–2Fe		63.8	918	36	
W–4Mo–8Ni–2Fe		63.8	947	31	
W–8Mo–8Ni–2Fe		65.9	1048	24	
W–12Mo–8Ni–2Fe		67.4	1119	14	
W–16Mo–8Ni–2Fe		68.4	1150	10	
W–2Mo–7Ni–3Fe	Conventional sintering 1500 °C for 30 min Heat treated at 1100 °C for 1 h and water-quenched	63	948	28	[57]
W–4Mo–7Ni–3Fe		64	980	24	
W–8Mo–7Ni–3Fe		68	1030	20	
W–12Mo–7Ni–3Fe		68	1100	10	
W–2Mo–7Ni–3Fe	SPS 1000–1250 °C For 8 min at 50 MPa 100 °C/min	63.9 at 1250 °C	390 bending strength at 1150 °C	—	[64]
W–7Ni–3Fe–4Mo	SPS 1100 °C For 8 min at 50 MPa 100 °C/min	286 HV	993	24	[65]
W–7Ni–3Fe–8Mo		336 HV	1050	20	
W–7Ni–3Fe–12Mo		354 HV	1120	10	
W–7Ni–3Fe–16Mo		372 HV	1150	7	
W–8Ni–2Fe–0Mo	High-energy ball milling for 40 h and SPS-sintered at 1000 °C for 8 min at 30 MPa and 100 °C/min	63	975	28	[66]
W–8Ni–2Fe–6Mo		65	1025	22	
W–8Ni–2Fe–12Mo		68	1120	14	
W–8Ni–2Fe–18Mo		72	1160	05	
W–8Ni–2Fe–24Mo		75	1250	02	

**Table 4:** Mechanical properties of W–Ni–Fe with lanthanum oxide.

Alloy	Sintering Process	Hardness (HRC)	Tensile Strength (MPa)	Elongation %	Reference
W–17.1Mo–2.5Ni-2Fe–0.2La–0.2Mn	Conventional sintering at 1510 °C for 90 min under a hydrogen atmosphere	34	650	2.2	[67]
W–16.9Mo–2.5Ni–2Fe–0.4La–0.2Mn	Conventional sintering at 1510 °C for 90 min under a hydrogen atmosphere	30	903	4.7	[67]
90W–7Ni–3Fe	Spark plasma sintering at 1100 °C for 5 min in a vacuum with a heating rate of 100 °C/min	138	475	0.64	[75]
W–7Ni–3Fe-0.50La_2_O_3_	Spark plasma sintering at 1100 °C for 5 min in a vacuum with a heating rate of 100 °C/min	370	822	0.95	[75]
W–7Ni–3Fe-1.00La_2_O_3_	Spark plasma sintering at 1100 °C for 5 min in a vacuum with a heating rate of 100 °C/min	533	1110	0.64	[75]

**Table 5:** Mechanical properties of W–Ni–Fe with yittrium and Y_2_O_3._

Alloy	Sintering Process	Hardness (HRB)	Tensile Strength (MPa)	Compression Strength (MPa)	Elongation (%)	Reference
W–5.6Ni–1.4Fe-00.1Y_2_O_3_	Sintering at 1485 °C for 60 min	—	828	—	14.6	[80]
W–5.6Ni–1.4Fe–0.1Y_2_O_3_	Sintering at 1485 °C for 120 min in a hydrogen atmosphere	—	883	—	18.4	[80]
W–7Ni–3Fe (Fine grained alloy)	Sintering at 1480 °C for 30 min in a hydrogen atmosphere	—	923	8	—	[82]
W–7Ni–3Fe–0.04Y_2_O_3_ (Fine grained alloy)		—	1050	30.8	—	[82]
W–4.9Ni–2.1Fe (Coarse grained)	Pre-sintering at 900 °C for 120 min and sintering at 1460 °C for 90 min in a hydrogen atmosphere	—	580 at 600 °C 330 at 800 °C	—	—	[83]
W–4.9Ni–2.1Fe–0.03Y (Fine grained)		—	620 at 600 °C 460 at 800 °C	—	—	[83]
W–7Ni–3Fe	Spark plasma sintering at 1100 °C with 30 MPa	68	586	—	0.64	[89]
W–7Ni–3Fe–0.25YSZ		109	892	—	1.83	
W–7Ni–3Fe–1YSZ		105	658	—	2.24	

## Data Availability

The data presented in this study are available on request from the corresponding author after obtaining permission of authorized person.
